# HIV-1 transgene expression in rats induces differential expression of tumor necrosis factor alpha and zinc transporters in the liver and the lung

**DOI:** 10.1186/1742-6405-8-36

**Published:** 2011-10-06

**Authors:** Pratibha C Joshi, David M Guidot

**Affiliations:** 1 Department of Medicine, Division of Pulmonary, Allergy, and Critical Care Medicine, Emory University School of Medicine, Atlanta, Georgia, USA; 2 The Atlanta VAMC, Decatur, Georgia, USA

**Keywords:** pulmonary, alveolar macrophages, AIDS, rodent, inflammation, micronutrients

## Abstract

**Background:**

Highly effective antiviral treatment can suppress HIV-1 infection, but the chronic effects of HIV-1-related viral proteins, including gp120 and Tat, on organs such as the lungs can be damaging. HIV-1 transgenic rodent models are useful for studying the systemic effects of these proteins independently of viral infection. We have previously shown that HIV-1 transgene expression (and therefore, HIV-1-related protein expression) in rats decreases alveolar macrophage zinc levels and phagocytic capacity by unknown mechanisms. We hypothesized that HIV-1 transgene expression induces chronic inflammation and zinc sequestration within the liver and thereby decreases zinc bioavailability in the lung. We examined the expression of the pro-inflammatory cytokine, tumor necrosis factor alpha (TNFα), the zinc storage protein, metallothionein (MT1), and the zinc exporter, ZNT1 in the livers and the lungs of wild type and HIV-1 transgenic rats ± dietary zinc supplementation. In addition, we measured zinc levels, the zinc importing protein ZIP1, and the phagocytic capacity in the alveolar macrophages.

**Results:**

HIV-1 transgene expression increased the liver-specific expression of TNFα, suggesting a chronic inflammatory response within the liver in response to HIV-1-related protein expression. In parallel, HIV-1 transgene expression significantly increased MT1 and ZNT1 expression in the liver as compared to the lung, a pattern that is consistent with zinc sequestration in the liver as occurs during systemic inflammation. Further, HIV-1 transgene expression decreased intracellular zinc levels and increased expression of ZIP1 in the alveolar macrophages, a pattern consistent with zinc deficiency, and decreased their bacterial phagocytic capacity. Interestingly, dietary zinc supplementation in HIV-1 transgenic rats decreased gene expression of TNFα, MT1, and ZNT1 in the liver while simultaneously increasing their expression in the lung. In parallel, zinc supplementation increased alveolar macrophage intracellular zinc levels and bacterial phagocytic capacity in HIV-1 transgenic rats.

**Conclusion:**

Taken together, these findings suggest that chronic HIV-1-related protein expression causes liver inflammation and zinc sequestration, which in turn limits zinc bioavailability in the lung and thereby impairs alveolar macrophage phagocytic function. Importantly, dietary zinc supplementation decreases liver inflammation and zinc sequestration and restores alveolar macrophage phagocytic function in HIV-1 transgenic rats, a result with potential clinical implications for improving lung health in HIV-1-infected individuals.

## Background

Despite significant advances in HIV-1 treatment including the development of highly active anti-retroviral therapy (HAART), infected individuals are prone to pneumonias by multiple pathogens including *Klebsiella pneumoniae *and *Mycobacterium tuberculosis *[1,2]. In addition, HIV-1-infected individuals are at increased risk for more typical community-acquired pneumonias from *Pneumococcal pneumoniae *and *influenza *[3,4]. Although the precise mechanisms are being investigated, HIV-1 can infect alveolar macrophages [5], and there is considerable evidence that phagocytosis and other innate immune functions are impaired in monocytes/macrophages from HIV-1 infected individuals [6,7]. For example, in immunocompetent individuals *Klebsiella pneumoniae *usually produces a mild respiratory illness. However, in alcoholics and in HIV-1-infected individuals, *Klebsiella *evades the normal innate immune defenses within the lower airways and can produce an overwhelming and frequently fatal pneumonia. In experimental models, HIV-1 infection decreases phagocytosis and increases the severity of lung infection from diverse organisms including *Streptococcus pneumoniae*, *Klebsiella pneumoniae*, *Mycobacterium tuberculosis*, and *Pneumocystis carinii *[1,2,6]. These observations raise the novel possibility that unrecognized macrophage dysfunction in HIV-1-infected individuals could contribute to the morbidity and mortality of lung infections in these vulnerable patients. Therefore, it is important to identify the mechanisms by which alveolar macrophage immune function is compromised in chronic HIV-1 infection so that new complementary therapies aimed at improving pulmonary host defenses can be developed.

Dietary zinc deficiency is common globally and appears to contribute to the pathogenesis of many diseases [8,9]. Besides dietary deficiency, low zinc levels are present in individuals with other abnormalities that either limit zinc absorption, such as in alcoholics, or that cause hepatic zinc sequestration, such as infections. While HIV-1 infection has a strong association with zinc deficiency, the exact mechanisms are not known. Zinc deficiency produces numerous abnormalities, but is particularly detrimental to epithelial cells and the immune system [9-11]. A higher incidence of bacterial infections was reported in HIV-1- infected persons with low zinc levels [12], and lower levels of zinc correlated with more advanced stages of the disease [13,14].

Transgenic models are useful to study the impact of HIV-1-related viral proteins and their roles in the pathogenesis of AIDS in tissues that are not directly infected with HIV-1 [15-17]. While such models have limitations [18], they have yielded significant insights into many aspects of HIV-1 pathogenesis. Transgenic mouse models have been instrumental in elucidating potential mechanisms underlying AIDS nephropathy [19] and cardiomyopathy [20]. Reid and colleagues established HIV-1 transgenic rat model in which affected animals are hemizygous for a NL4-3Δ *gag/pol *transgene [21]. As a consequence of expressing HIV-1 proteins such as gp120, Nef, Tat, and Rev, these transgenic rodents develop muscle wasting, cataracts, nephropathy, skin lesions and immune deficiencies that are remarkably similar to the manifestations of AIDS in humans [21,22]. We recently reported that these HIV-1 transgenic rats have circulating levels of gp120, and that the alveolar zinc concentrations decrease with age [23]. The expression of granulocyte-macrophage colony stimulating factor (GM-CSF) receptors on alveolar macrophages from HIV-1 transgenic rats was also significantly decreased [23], and was associated with impaired macrophage phagocytic function. Interestingly, treating alveolar macrophages from HIV-1 transgenic rats with zinc acetate *in vitro *improved their bacterial phagocytic function [23]. However, in that study we did not examine the potential mechanism(s) by which HIV-related protein expression decreases zinc bioavailability within the lung.

Organ interactions, including liver-lung interactions, modulate inflammatory and immune responses during illness [24]. In particular, there is evidence that during inflammation the liver sequesters zinc, perhaps as an adaptive response to limit zinc bioavailability to pathogenic microbes [25,26]. However, if inflammation persists and hepatic zinc sequestration continues, zinc bioavailability to other tissues such as the lung could be compromised. Therefore, we hypothesized that the zinc deficiency and consequent epithelial and macrophage dysfunction we previously identified in the lungs of HIV-1 transgenic rats [23,27] might be caused, at least in part, by chronic liver inflammation and consequent changes in zinc storage proteins that mediate zinc sequestration in the liver. To test this hypothesis, we compared the relative expression of the stereotypical pro-inflammatory cytokine, tumor necrosis factor α (TNFα), in the livers and in the lungs of HIV-1 transgenic rats and in their wild type littermates. In parallel, we compared the relative expression of key zinc storage and transport proteins in the livers and in the lungs of these animals. Finally, we determined the effects of dietary zinc supplementation on liver inflammation and on alveolar macrophage zinc bioavailability and bacterial phagocytic function, with the rationale that such a strategy could be used to improve lung health in individuals infected with HIV-1.

## Materials and methods

### HIV-1 transgenic rats

Age-matched HIV-1 transgenic or wild type male Fischer 344 rats were either purchased from Harlan Laboratories or produced by a breeding colony we established. This transgenic rat strain was genetically engineered to contain the entire genome of the HIV-1 virus except that the 3' region of gag and the 5' region of pol are deleted [23]. These transgenic rats are born with cataracts and can be clearly distinguished from wild type littermates by this phenotype and by tail-snip DNA analysis. All the work was performed under the approval of the Institutional Animal Care and Use Committee at the Atlanta VA Medical Center. In most experiments, we used transgenic and gender-matched wild type littermates between the ages of 8-9 months. Zinc acetate (100 mg/L) was added to the liquid diets of some rats for 8 weeks prior to sacrifice.

### RNA isolation and real-time PCR

Total RNA was extracted using Qiagen RNeasy Mini Kit (Valencia, CA) and reverse transcription was performed with Bio-Rad iScript cDNA synthesis kit (Hercules, CA). Real-time PCR was carried on the Bio-Rad iCycler. Amplification was performed in Bio-Rad iQ SYBR green supermix containing specific primers and with denaturing at 95^°^C for 20s, annealing at 58^°^C for 20s, and extension at 72^°^C for 20s. Standards and samples were run in triplicate. The primers were designed in our laboratory and were obtained from Invitrogen (Carlsbad, CA). QuantumRNA class II 18S primers were purchased from Ambion (Austin, TX). PCR amplicons from all species were normalized for the amount of 18S in the same cDNA sample. Real time SYBR green dissociation curves showed one species of amplicon for each primer set.

### Bronchoalveolar lavage and isolation of alveolar macrophages

Following pentobarbital anesthesia (100 mg/kg IP), a tracheotomy tube was placed and rat lungs were lavaged × 4 with 10 ml of sterile cold PBS (pH 7.4). The recovered lavage solution was centrifuged at 1500 rpm for 7 min, and the cell pellet re-suspended in sterile media for functional studies. This procedure routinely yields cells that are >98% viable by trypan blue exclusion test. Diff-Quick (IMEB, INC) and CD-68 staining is used to show that >95% cells are alveolar macrophages. For selected analyses of the effects of zinc depletion on the expression of zinc transporters in macrophages, we used the rat alveolar macrophage cell line, NR8383 (ATCC, Manassas, VA), were maintained in F12K culture media with 15% fetal bovine serum.

### Flow cytometric detection of intracellular zinc

Zinc levels in the freshly isolated alveolar macrophages were measured using the zinc-specific dye, FluoZin-3AM (Invitrogen), a recently developed fluorochrome with a high affinity for zinc (K_d _15 nM). This is a membrane-permeable dye used to detect intracellular zinc. The alveolar macrophages were stained with this dye for 1 hr at room temperature, and the resulting fluorescence was measured using the FACScan Flow Cytometer (Becton Dickinson). The data are expressed as % positive cells.

### Flow cytometric detection of MT1 expression

The intracellular expression of MT1 in the freshly isolated rat alveolar macrophages was measured by an established protocol [23]. Briefly, cells were made permeable with 0.1% saponin in PBS followed by incubation with rabbit anti-MT1 antibody (Santa Cruz Biotechnology, Santa Cruz, CA) or an isotype-matched control antibody for 30 minutes at room temperature. Cells were washed with PBS-saponin before adding FITC-conjugated donkey anti-rabbit secondary antibody (Santa Cruz Biotechnology, Santa Cruz, CA). Cells were washed with PBS and analyzed by FACScan Flow Cytometer (Becton Dickinson).

### Macrophage phagocytosis in vitro

Freshly isolated rat alveolar macrophages were incubated for 1 hour with FITC-conjugated *Staphylococcus aureus *(Wood Strain without protein A; Molecular Probes, Eugene, OR) in a 10:1 ratio. Cells were vigorously washed × 3 with PBS and extracellular fluorescence was quenched by adding trypan blue [23]. Cells with FITC-conjugated bacteria were measured by flow cytometry. Phagocytic index was calculated as follows: (% positive cells × mean channel fluorescence)/100.

### Statistical analyses

Data are presented as mean ± SEM. Data analysis was done by one-way ANOVA with Student-Newman-Keuls test for group comparison, and differences among groups were considered statistically significant at a p value of <0.05.

## Results

### HIV-1 transgenic rats are smaller than their wild type littermates

Our previous studies have provided new evidence for the effects of HIV-1- related proteins in the alveolar space that could have implications for understanding why HIV-1-infected individuals are prone to pulmonary infections and respiratory failure [23,27]. Consistent with the conclusion that not all of the systemic manifestations of AIDS can be attributed directly to viral infection and replication within target tissues (many of which cannot be infected by the virus), by the age of 7 months the HIV-1 transgenic rats begin to display modestly decreased body weights compared to wild type littermates. By the age of 9 months they are clearly smaller than their wild type littermates and exhibit systemic signs of illness including poor grooming and decreased physical activity (Additional File 1 Figure 1, panels A&B). By 12 months of age the HIV-1 transgenic rats show signs of severe disease including hind limb atrophy and gait difficulties (not shown). These observations are in agreement with those of Reid and colleagues [21] who developed this model, and confirm that chronic HIV-1-related protein expression causes a progressive systemic phenotype that resembles AIDS in humans. These findings are consistent with the extensive experimental and clinical evidence that these HIV-1-related proteins are themselves toxic to a variety of target tissues that are not infected directly by HIV-1.

**Figure 1:**
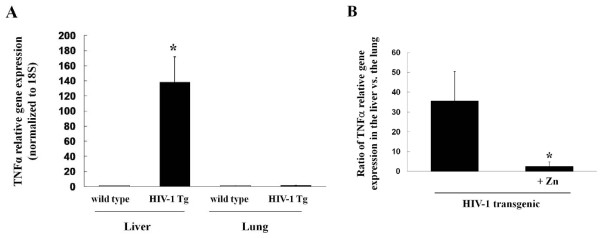
**HIV-1 transgene expression significantly increases the expression of the pro-inflammatory cytokine, TNFα, in the liver but not in the lung, and this pro-inflammatory effect is reversed by dietary zinc supplementation**. Panel A shows the relative gene expression of TNFα in the liver and the lung of wild type and transgenic rats. * p < 0.05 increased compared to liver expression in wild type rats. Panel B shows the effects of dietary zinc supplementation (100 mg/L) on the ratio of relative TNFα expression in the liver vs. the lung in HIV-1 transgenic rats. * p < 0.05 decreased compared to untreated HIV-1 transgenic rats (no zinc supplements). In all determinations shown in Panel A and B, RNA was extracted from the tissues and converted to cDNA. Relative gene expression of TNFα was determined by real-time PCR using 18S as control and calculated by the ΔΔCT method. Each value represents the mean ± SEM of 4-6 determinations.

### Increased expression of the pro-inflammatory cytokine TNFα in the livers of HIV-1 transgenic rats suggests a chronic inflammatory response

During the acute phase reaction associated with infections, there is typically an increase in pro-inflammatory cytokine expression in the liver. As shown in Figure 1 (panel A), gene expression of TNFα in the liver, but not in the lung, is highly up-regulated in HIV-1 transgenic rats, suggesting a chronic inflammatory response within the liver. Interestingly, dietary zinc supplementation (panel B) significantly attenuated TNFα expression in the liver such that the relative expression in the liver vs. the lung decreases from ~35:1 to ~2.5:1. These results suggest that the chronic and systemic expression of HIV-1-related proteins, such as occurs during HIV-1 infection, causes a pro-inflammatory response within the liver, but that the provision of high levels of dietary zinc somehow mitigates this liver inflammation.

### Increased expression of MT1 and ZNT1 in the livers of HIV-1 transgenic rats suggests zinc sequestration

**Figure 2:**
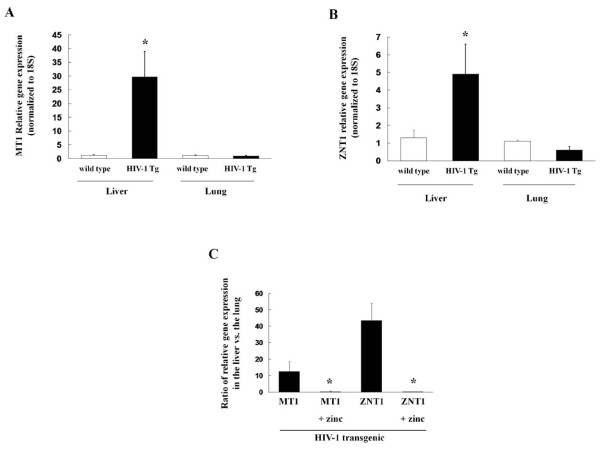
**HIV-1 transgene expression alters the gene expression of the zinc storage protein metallothionein-1 (MT1) and the zinc efflux transporter ZNT1 in the liver and the lung, and these effects are also reversed by dietary zinc supplementation**. Panels A&B show the relative gene expression of MT1 and ZNT1, respectively in the liver and the lung of wild type and transgenic rats. *p < 0.05 increased compared to liver expression in wild type rats. Panel C shows the effects of dietary zinc supplementation (100 mg/L) on the ratio of relative gene expression of MT1 and ZNT1 in the liver and the lung of HIV-1 transgenic rats. *p < 0.05 decreased compared to untreated HIV-1 transgenic rats (no zinc supplements). In all determinations shown in Panels A, B and C, RNA was extracted from the tissues and converted to cDNA. Relative gene expressions of MT1 and ZNT1 were determined by real-time PCR using 18S as control and calculated by the ΔΔCT method. Each value represents the mean ± SEM of 4-6 determinations.

In the acute phase reaction the liver is known to sequester zinc. Although our HIV-1 transgenic rat model is not an infectious model, HIV-1 transgenic rats show signs that are typical for chronic infections including cachexia and, as noted above, a dramatic increase in liver-specific expression of TNFα. Therefore, we examined two indicators of zinc status in the livers of wild type and HIV-1 transgenic rats. Metallothionein 1 (MT1) is a cysteine-rich protein that binds zinc and is usually increased when zinc levels are high. In parallel, zinc transporters tightly regulate the movement of zinc across plasma and organellar membranes, and are sensitive indicators of zinc bioavailability at the cellular level. The solute carrier-39 (SLC39) transporter family includes a subfamily of ZRT/IRT-related proteins (ZIPs) that import zinc into the cytosol [28,29]. Another family of transporters, the solute carrier-30 (SLC30) or Cation Diffusion Facilitator (CDF) [28,29], include the ZNT subfamily that export zinc from the cytosol. Zinc exporters (ZNTs) are up-regulated in cells when zinc levels are high (to increase export and therefore prevent intracellular zinc toxicity). As shown in Figure 2 (panels A&B), liver and lung tissues were probed by real-time PCR for the expression of the zinc storage protein, MT1 and the zinc exporter, ZNT1. The increase in MT1 and ZNT1 in the livers of HIV-1 rats was about 39-fold and 11-fold, respectively, compared to that of wild type rats; this pattern is consistent with hepatic zinc sequestration (i.e. high tissue zinc levels). Interestingly, dietary zinc supplementation decreased the liver-specific expression of MT1 and ZNT1, paralleling its effects on TNFα expression and consistent with the explanation that the observed inflammation was causing the liver to sequester zinc. In contrast, the lung-specific expression of MT1 and ZNT1 was increased after zinc supplementation, indicating increased zinc bioavailability to the lung. The ratios of the relative gene expressions of MT1 and ZNT1 in the liver vs. the lung are shown in Figure 2, panel C. Taken together, the data presented in Figures 1 and 2 suggest that HIV-1-related protein expression produces a state of chronic liver inflammation that promotes hepatic zinc sequestration and, as a consequence, limits zinc bioavailability to other organs such as the lung. Although as yet unexplained, the provision of high levels of zinc as a dietary supplement somehow inhibits the aberrant expression of TNFα and associated zinc sequestration in the liver and increases zinc bioavailability in the lungs of HIV-1 transgenic rats.

### HIV-1 transgene expression increased gene expression of ZIP4 (a zinc importer) and decreased the expression of ZNT4 (another zinc exporter) in the alveolar macrophages, and these effects can be reproduced by treating a macrophage cell line with a zinc chelator *in vitro*

**Figure 3:**
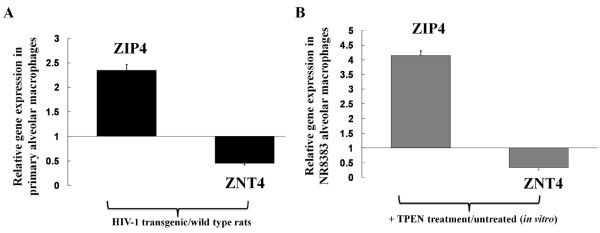
**HIV-1 transgene expression, or acute zinc chelation with TPEN, alters gene expression of the zinc transporters ZIP4 and ZNT4 in alveolar macrophages**. Panel A shows the relative expression of ZIP4 and ZNT4 in alveolar macrophages isolated from HIV-1 transgenic rats as compared to alveolar macrophages isolated from wild type rats. Panel B shows the relative expression of ZIP4 and ZNT4 in the rat alveolar macrophage cell line NR8383 (ATCC) treated with or without the zinc chelator TPEN (5 μM) *in vitro *for 24 h. In both panels, gene expression by real time PCR was calculated by the ΔΔCT method and the expression in HIV-1-derived macrophages (Panel A) and TPEN-treated NR8383 cells (Panel B) were normalized to the expression for macrophages from wild type rats (panel A) and untreated NR8383 cells (panel B). Each value represents the mean ± SEM of 4 determinations.

As HIV-1 transgene expression significantly lowers zinc levels in the alveolar space, we next examined its effects on the expression of zinc transporters in the alveolar macrophages. Data are presented in Figure 3 (panel A) as the expression of ZIP4 and ZNT4 in HIV-1-derived macrophages when normalized to the expression of macrophages from wild type rats. This expression pattern is consistent with a state of zinc deficiency (i.e., increase in zinc importers and a decrease in zinc exporters in an effort to preserve intracellular zinc pools). Further evidence that these changes in ZIP4 and ZNT4 expression reflect zinc deficiency is provided in panel B. In these experiments, the rat alveolar macrophage cell line, NR8383 was treated *in vitro *with or without the zinc chelator, N,N,N',N'-tetrakis-(2-pyridyl-methyl) ethylenediamine (TPEN, 5μM) for 24 h, at which time ZIP4 and ZNT4 expression were determined. As is evident in panel B, acute zinc deficiency induced by TPEN treatment caused changes in ZIP4 and ZNT4 expression that were comparable to those seen in the alveolar macrophages of HIV-1 transgenic rats (panel A).

### HIV-1 transgene expression decreased metallothionein expression and zinc levels in the alveolar macrophage and these changes can be rapidly reversed with zinc treatment

**Figure 4:**
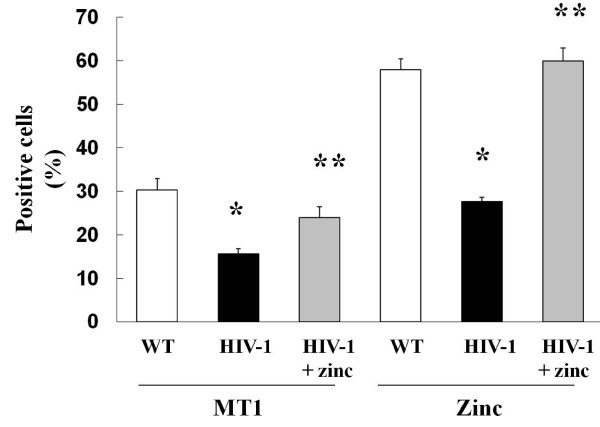
**HIV-1 transgene expression decreases metallothionein expression and zinc levels in alveolar macrophages and these changes can be rapidly reversed with dietary zinc supplementation**. Shown are the percentage of alveolar macrophages that were positive for MT1 (left three columns) and for zinc (right three columns) by flow cytometry performed on macrophages isolated from wild type rats (WT), from HIV-1 transgenic rats (HIV-1) and from HIV-1 transgenic rats whose drinking water was supplemented with 100 mg/L of zinc acetate (HIV-1 + zinc). HIV-1 transgenic rats have significantly decreased MT1 expression and intracellular levels of zinc in their alveolar macrophages. Each value represents the mean ± SEM of 4 determinations. *p < 0.05 decreased compared to untreated wild type rats. **p < 0.05 increased compared to untreated HIV-1 transgenic rats.

As discussed, the zinc storage protein metallothionein is a good indicator of zinc status in cells and tissues [30]. Since intracellular zinc levels were significantly decreased in transgenic rats [9,23], we examined MT1 expression in the alveolar macrophages from wild type and HIV-1 transgenic rats. As predicted, flow cytometric analysis of alveolar macrophages from HIV-1 transgenic rats showed a significant decrease in MT1 and zinc positive cells (Figure 4), which is consistent with our previously reported findings [23]. Importantly, supplementing the diet with zinc restored intracellular zinc and MT1 levels (Figure 4).

### Dietary zinc supplementation *in vivo *restores phagocytic function of alveolar macrophages from HIV-1 transgenic rats

**Figure 5:**
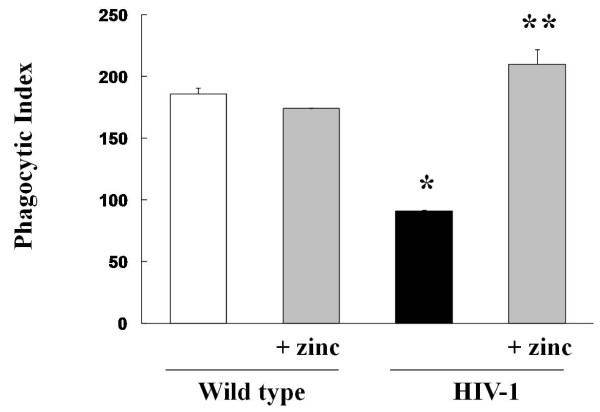
**Dietary zinc supplementation in HIV-1 transgenic rats increases the bacterial phagocytic capacity of alveolar macrophages**. The bacterial phagocytic capacity (as reflected by phagocytic indices) of alveolar macrophages from wild type and HIV-1 transgenic rats were determined by flow cytometry as described in Materials and Methods. Each value represents the mean ± SEM of 4-6 rats in each group. *p < 0.05 decreased compared to untreated wild type rats. **p < 0.05 increased compared to untreated HIV-1 transgenic rats.

HIV-1 transgenic rats have significantly decreased intracellular levels of zinc in their alveolar macrophages as compared to cells from age-matched wild type rats [23]. In this study, no increase in intracellular zinc was observed in wild type rats after dietary zinc supplementation. In contrast, HIV-1 transgenic rats whose diets were supplemented with zinc had significantly higher intracellular zinc levels in their alveolar macrophages (Figure 4B), indicating that these cells can indeed import zinc *in vivo *if alveolar levels are sufficient. We have previously determined that alveolar macrophages from HIV-1 transgenic rats had decreased bacterial phagocytic capacity [23] and this finding was confirmed in the current study as shown in Figure 5. In contrast, dietary zinc supplementation not only increased cellular zinc levels, it also restored bacterial phagocytic capacity in the alveolar macrophages of HIV-1 transgenic rats (Figure 5). These new findings extend our previous study in which we determined that treating alveolar macrophages from HIV-1 transgenic rats with zinc acetate *in vitro *restored their bacterial phagocytic capacity and raise the possibility that dietary zinc supplementation could improve alveolar macrophage function in HIV-1-infected individuals. It is important to note that we did not identify any effects of dietary zinc supplementation on alveolar macrophages from wild type rats in terms of zinc levels (not shown) or in bacterial phagocytic capacity (Figure 5). This is consistent with the known mechanisms by which cellular zinc levels are maintained in an optimal range by this sensitive transporter system that senses cellular zinc requirements and regulates the import and export of zinc in an efficient manner as long as zinc bioavailability is not limited within the cellular microenvironment (such as the alveolar space).

## Discussion

In this study we determined that chronic HIV-1 transgene expression in rats, which causes a progressive systemic illness that recapitulates many of the manifestations of AIDS in humans, increases the expression of the pro-inflammatory cytokine TNFα in the liver but not in the lung. In parallel, this chronic and systemic expression of HIV-1-related proteins alters the expression of zinc transporters and storage proteins within the liver in a pattern that is consistent with hepatic zinc sequestration. In contrast, zinc bioavailability in the lung and the alveolar space is markedly decreased, and alveolar macrophages become zinc-deficient despite altering their cell-specific expression of zinc transporters in a pattern that promotes cellular zinc import and retention. This apparent hepatic zinc sequestration, and consequent relative zinc deficiency within the alveolar space, has profound consequences on alveolar macrophage bacterial phagocytic capacity. In contrast, HIV-1 transgenic rats whose diets were supplemented with zinc had no evidence of liver inflammation or zinc sequestration and, in parallel, had the same alveolar macrophage phagocytic function as their wild type littermates. Taken together, these results suggest a novel mechanism by which chronic HIV infection may cause alveolar macrophage immune dysfunction and raise the possibility that dietary zinc supplementation could enhance pulmonary host defenses in these vulnerable individuals.

**Figure 6:**
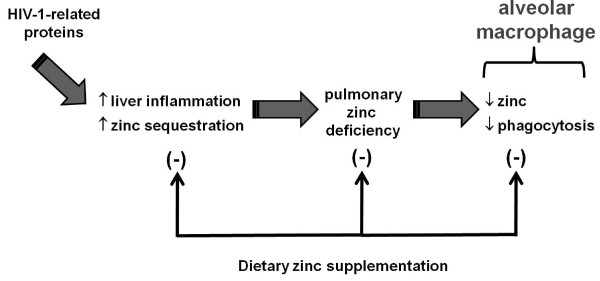
**Proposed hypothetical scheme: HIV-1 transgene and proteins cause hepatic inflammation and hepatic zinc sequestration resulting in pulmonary zinc deficiency, and decreased alveolar macrophage function**. Dietary zinc supplementation modulates this vicious cycle and reduces pulmonary effects mediated by HIV-1 transgene and related proteins.

This hypothetical scheme is depicted in Figure 6. We speculate, based on our previous and current studies, that the systemic expression of HIV-1-related proteins, as occurs during chronic HIV-1 infection, promotes both hepatic inflammation and zinc sequestration. As a consequence, extrahepatic tissues including the lung become relatively zinc-deficient, and this has serious consequences for alveolar macrophage immune function. Importantly and with potential clinical therapeutic interventions, dietary zinc supplementation somehow mitigates these pathophysiological effects in the liver and restores alveolar macrophage zinc bioavailability and bacterial phagocytic function. If these experimental findings ultimately translate to the human condition, then the relatively simple use of dietary zinc supplementation could be an effective adjunctive therapy in HIV-1-infected individuals.

To our knowledge this is the first experimental evidence that systemic expression of HIV-1-related proteins causes these differential effects on inflammation and zinc bioavailability in the liver vs. the lung. Therefore, we believe these findings are provocative in that they provide a novel mechanism by which chronic HIV-1 infection could impair innate immunity within the lower airways. However, there are some limitations to this initial study that will require future studies to address. Specifically, it is as yet unknown how these HIV-1-related proteins promote TNFα expression within the liver and not in the lung. Perhaps even more important to determine is how dietary zinc supplementation mitigates liver-specific inflammation and apparent zinc sequestration. As zinc is a cofactor for more than 300 enzymes and is critical to the function of thousands of transcription factors, its efficacy in this context is likely attributable to more than one specific effect. Further, the salutary effects of dietary zinc supplementation on alveolar macrophage zinc bioavailability and phagocytic function may not occur independently of, or in spite of, the HIV-1-induced liver inflammation and zinc sequestration, as zinc corrects both the liver and lung abnormalities. Therefore, the provision of dietary zinc supplements (at levels that are many times higher than could possibly be contained in even the 'healthiest' of diets), somehow mitigates the liver inflammation and zinc sequestration and may thereby make zinc available to extrahepatic tissues including the lung. Specifically, if dietary zinc supplements were simply bypassing the liver and replenishing the lung's supply, this might explain the beneficial effects on alveolar macrophage function but would not explain the remarkable reversal of liver-specific TNFα expression and the associated changes in zinc storage and transport proteins that suggest hepatic zinc sequestration.

Despite improvements in HIV-1 treatment there are compelling reasons to identify and test such adjunctive treatments, as pulmonary infections and respiratory failure remain significant challenges even in the era of anti-retroviral therapy and are associated with high rates of morbidity and mortality. Therefore, although the overall prognosis has greatly improved it remains critically important to understand how HIV-1 renders the lung susceptible to infection and injury. Evidence continues to evolve implicating HIV-1-related proteins, including gp120 and Tat, as mediators of oxidant stress and injury even in target cells that are not directly infected with the virus. We had previously reported that this may be due in part to a significant decrease in the levels of the antioxidant glutathione in the alveolar space [27]. Further, we have identified that these proteins cause significant zinc deficiency within the lung, and zinc is essential for both immune and anti-oxidant defenses. This may be particularly relevant during chronic malnutrition where glutathione and zinc decrease during starvation or protein-deficiency [30-33]. However, in our study rats were pair-fed a protein-sufficient diet and there were no significant differences in the consumption of diet between wild type and transgenic rats. Therefore, it is plausible to speculate that chronic expression and circulation of HIV-1-related proteins causes liver inflammation (as reflected by high TNFα expression) and malaise. The liver is the primary source of glutathione synthesis and storage in the body and, as zinc is known to restore glutathione levels in cells [34], it is possible that dietary zinc supplementation decreased liver inflammation in HIV-1 transgenic rats at least in part by augmenting glutathione levels in the liver. Regardless of the specific mechanism(s) involved, the effects of dietary zinc supplementation on hepatic TNFα expression were remarkable. Further, the down-modulation of hepatic inflammation reversed the changes in the expression of zinc storage and transport proteins that promote zinc sequestration and restored zinc bioavailability and phagocytic function to the alveolar macrophage. Although the underlying mechanisms will need to be dissected, these findings provide intriguing evidence that HIV-1-related proteins can impact lung host immunity by an unexpected effect on hepatic inflammation and zinc sequestration. In this context, our current findings also complement our previous studies in which we determined that HIV-1 transgene expression causes oxidative stress, glutathione depletion, and alveolar epithelial barrier dysfunction [27].

These effects on alveolar host defenses and the epithelium are clinically relevant, as a robust response to environmental challenges including microbial invasion of the lower airways requires coordinated defenses involving the alveolar epithelial barrier and the resident alveolar macrophage. We have taken advantage of this novel HIV-1 transgenic rat model, in which HIV-1-related proteins are expressed and the animal develops an impressive AIDS-like phenotype in the absence of viral infection/replication, to study the effects of these proteins on alveolar macrophage and epithelial function. Although anti-retroviral therapy can control viral replication in most cases, the HIV-1 related proteins still circulate [35] and can have adverse effects on cellular function. The data presented here suggest that supplementing anti-retroviral therapy with dietary zinc could be beneficial to pulmonary host immune responses. Further, such a complementary therapy could also benefit HIV-infected patients with co-morbid conditions such as alcohol abuse, as we recently determined that dietary zinc supplementation also improves alveolar macrophage function in alcohol-fed rats [36].

## Conclusion

In summary, we report that HIV-1 transgene expression, and therefore the chronic expression and systemic circulation of HIV-1-related proteins, induces an acute phase-like response resulting in a differential expression of TNFα in the liver as compared to the lung. In parallel, alterations in the expression of zinc storage and transport proteins suggest zinc sequestration in the liver and as a consequence, zinc deprivation and alveolar macrophage dysfunction in the lung. In contrast, dietary zinc supplementation decreases TNFα expression and apparent zinc sequestration in the liver, replenishes intracellular levels of zinc in the alveolar macrophages, and improves alveolar macrophage bacterial phagocytic function.

## Abbreviations

GM-CSF: granulocyte-macrophage colony stimulating factor; HAART: highly active anti-retroviral therapy; MT1: metallothionein 1; TNFα: tumor necrosis factor alpha; TPEN: N,N,N',N'-tetrakis-(2-pyridyl-methyl) ethylenediamine; ZIP: ZRT/IRT-related proteins

## Competing interests

The authors declare that they have no competing interests.

## Authors' contributions

PCJ: conception and design, data collection and analysis, figure and manuscript preparation; DMG: design, intellectual content, and editorial support All authors have read and approved the final manuscript.

## Supplementary Material

Additional file 1 **Body weights of wild type and HIV-1 transgenic rats**. Panel A shows representative pictures of these rats in each group; of particular note are the smaller body size and the scruffy coat in the HIV-1 transgenic rat compared to its wild type littermate. Panel B shows the body weights of 9 month old wild type (WT) and HIV-1 transgenic (HIV-1-Tg) littermate rats; each value represents the mean ± SEM of 6 rats. * p < 0.05 decreased body weights compared to wild type littermates. Click here for file
