# Supplementary figures and images for: HIV-1 transgene expression in rats induces differential expression of tumor necrosis factor alpha and zinc transporters in the liver and the lung
Source: AIDS Res Ther. 2011 Oct 6;8:36. doi: 10.1186/1742-6405-8-36 (PMC3204218; doi:10.1186/1742-6405-8-36)

## Slide 1
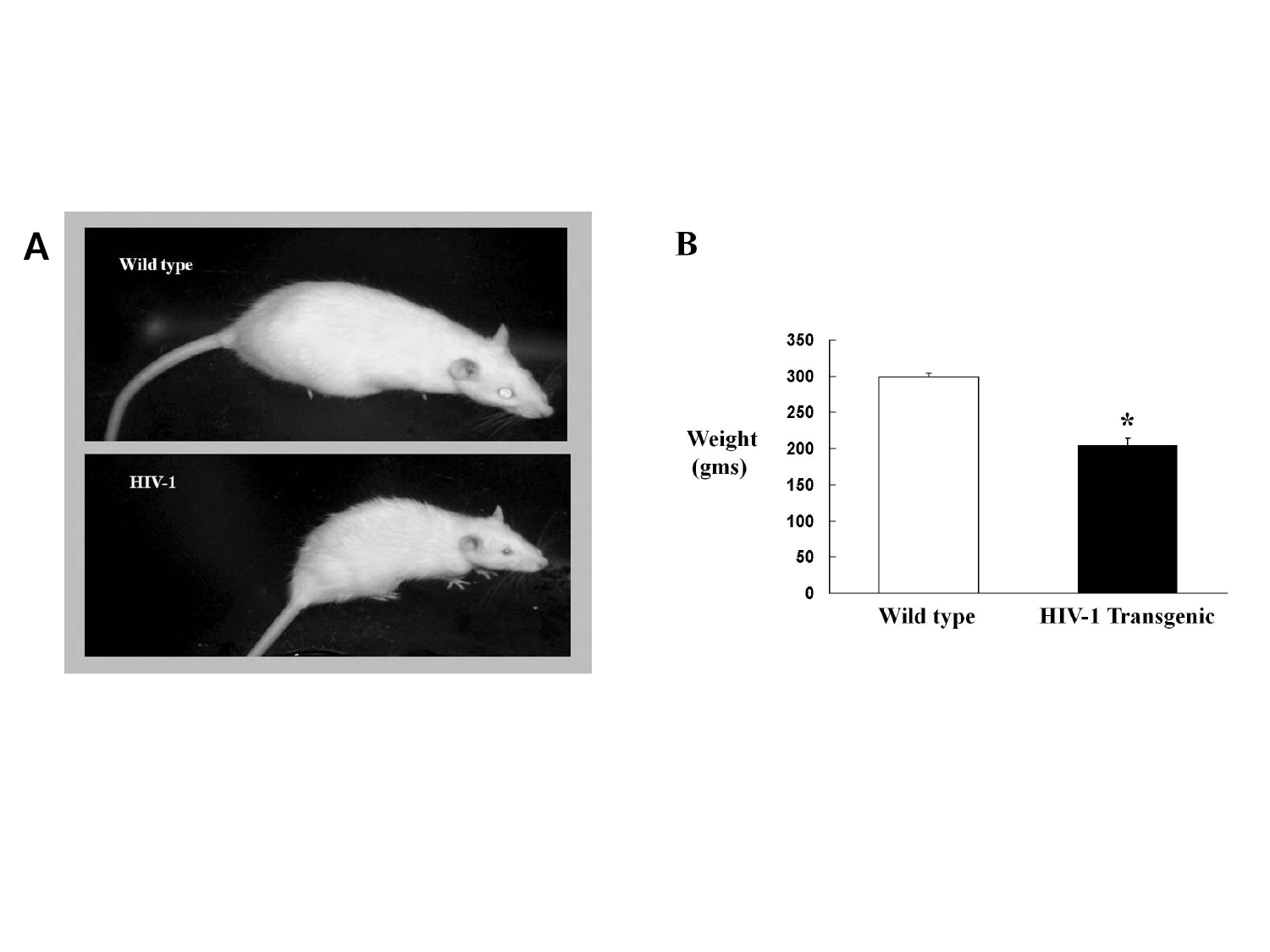

A

Supplement: Additional file 1 — Body weights of wild type and HIV-1 transgenic rats. Panel A shows representative pictures of these rats in each group; of particular note are the smaller body size and the scruffy coat in the HIV-1 transgenic rat compared to its wild type littermate. Panel B shows the body weights of 9 month old wild type (WT) and HIV-1 transgenic (HIV-1-Tg) littermate rats; each value represents the mean ± SEM of 6 rats. * p < 0.05 decreased body weights compared to wild type littermates. [file 1742-6405-8-36-S1.PPT]
